# Meta-Analysis of the Association between Vitamin D Receptor Polymorphisms and the Risk of Autoimmune Thyroid Disease

**DOI:** 10.1155/2018/2846943

**Published:** 2018-03-22

**Authors:** Xue-Ren Gao, Yong-Guo Yu

**Affiliations:** Department of Pediatric Endocrinology/Genetics, Shanghai Institute for Pediatric Research, Xinhua Hospital, School of Medicine, Shanghai Jiao Tong University, Shanghai 200092, China

## Abstract

The association between vitamin D receptor (*VDR*) polymorphisms (rs731236, rs1544410, rs2228570, and rs7975232) and the risk of autoimmune thyroid disease (AITD) had been investigated in previous studies. However, the results of these studies remained controversial. Thus, a meta-analysis was performed to derive a more precise conclusion. All related articles were systematically searched by PubMed, Embase, Google Scholar, and Chinese National Knowledge Infrastructure (CNKI). The pooled odds ratios (ORs) with 95% confidence intervals (CIs) were calculated to assess the strength of association. The overall results indicated that *VDR* rs731236 and rs2228570 polymorphisms were significantly associated with a reduced risk of AITD. However, a stratification analysis based on clinical types showed that *VDR* rs731236 and rs2228570 polymorphisms were associated only with a reduced risk of HT. A stratification analysis by ethnicity showed that *VDR* rs731236 polymorphism was significantly associated with a reduced risk of AITD in Asian and African populations. *VDR* rs2228570 polymorphism was associated with a reduced risk of AITD in Asian populations. *VDR* rs1544410 polymorphism was associated with a reduced risk of AITD in European and African populations, but with an increased risk of AITD in Asian populations. *VDR* rs7975232 polymorphism was significantly associated with an increased risk of AITD in African populations. In conclusion, the present study suggested that *VDR* rs731236, rs1544410, rs2228570, and rs7975232 polymorphisms were significantly associated with AITD risk. However, more well-designed studies should be performed to verify the current results.

## 1. Introduction

Autoimmune thyroid disease (AITD), mainly including Graves' disease (GD) and Hashimoto's thyroiditis (HT), is an organ-specific autoimmune disease and affects up to 5% of the general population [1, 2]. Although the pathogenesis of AITD is still unknown, it is generally acknowledged that environmental factors and the intrinsic genetic predisposition of an individual play critical roles in the occurrence of the disease [3]. Environmental factors, such as viral infections, irradiation, drugs, and iodine intake, may involve interference with thyroid function, direct toxic effects on thyrocytes, immune stimulation, or other immunomodulatory effects [3, 4]. However, it is difficult to directly link an environmental exposure with AITD due to the intervention of genetic factors. Increasing evidence suggests that single-nucleotide polymorphisms (SNPs) in AITD-related genes can influence individual predisposition to the disease [5–7].

Vitamin D is a fat-soluble vitamin and is activated in the liver and kidney [8]. Activated vitamin D [1,25(OH)_2_D] can promote the differentiation of monocytes and inhibit the maturation of dendritic cells [9]. Furthermore, it can also suppress the production of cytokines, such as interleukin-1, interleukin-2, interleukin-6, and tumor necrosis factor [10]. These cytokines play important roles in the development of lymphocytes, which are believed to be involved in the pathogenesis of autoimmune diseases. The immunomodulatory actions of 1,25(OH)_2_D are mediated by its binding to vitamin D receptor (VDR), which belongs to the family of *trans*-acting transcriptional regulatory factors and is widely expressed in various immune cell subsets, including lymphocytes, macrophages, and several endocrine cells [11]. The gene encoding VDR contains 14 exons and spans approximately 75 kilobases on chromosome 12q13.11. Many SNPs have been identified in the *VDR* gene [12]. Among them, the association of *VDR* rs731236, rs1544410, rs2228570, and rs7975232 polymorphisms with AITD risk has been widely reported [13–34]. However, the results are inconsistent and ambiguous. Furthermore, considering that a single-center pilot study with small sample sizes may possess low statistical power, we performed a meta-analysis of all eligible studies to obtain a more precise conclusion.

## 2. Methods

### 2.1. Search Strategy

All related articles were obtained by systematically searching PubMed, Embase, Google Scholar, and Chinese National Knowledge Infrastructure (CNKI). The search keywords were as follows: “vitamin D receptor OR VDR,” “polymorphism OR genetic variation OR genetic variant,” and “autoimmune thyroid disease OR AITD OR thyroid.” There were no limitations on language and year of publication. The last search was updated on August 28, 2017. Furthermore, the references of all related articles were also retrieved to find other eligible studies.

### 2.2. Inclusion and Exclusion Criteria

All eligible studies must meet the following inclusion criteria: (a) case-control studies; (b) evaluation of the association between *VDR* polymorphisms (rs731236, rs1544410, rs2228570, and rs7975232) and AITD risk; and (c) available genotype/allele frequencies. In addition, the exclusion criteria were as follows: (a) letters, reviews, and case reports and (b) duplicate publication. If multiple studies had overlapping data, only those with complete data were included.

### 2.3. Data Extraction

Two authors independently reviewed the related articles and extracted the following data: first author's name, year of publication, region, ethnicity, genotyping methods, the number of cases and controls, and genotype/allele frequency. Any disagreement was resolved by discussion with each other.

### 2.4. Statistical Analysis

Hardy-Weinberg equilibrium (HWE) in the control group of each study was calculated by chi-square goodness-of-fit test, and *P*_HWE_ < 0.05 was considered as a deviation from HWE. The strength of the association between *VDR* polymorphisms (rs731236, rs1544410, rs2228570, and rs7975232) and AITD risk was evaluated by the pooled odds ratios (ORs) with 95% confidence intervals (CIs). The significance of the pooled ORs was assessed by the *Z* test, and *P*_*Z*_ < 0.05 was considered statistically significant. The chi-square based *Q*-test was used to investigate the between-study heterogeneity. If *P*_H_ < 0.1 indicated the existence of between-study heterogeneity, the random-effect model was used to calculate the pooled ORs; otherwise, the fixed-effect model was applied for the analysis. A sensitivity analysis was conducted by omitting one study each time to estimate the stability of the result. Publication bias was determined by Begg's funnel plot and Egger's test. A symmetric funnel plot and *P* value of Egger's test more than 0.05 indicated the lack of publication bias. All statistical tests were performed using Review Manager 5.2 (The Nordic Cochrane Centre, The Cochrane Collaboration, Copenhagen) and the STATA 12.0 software (Stata Corporation, College Station, TX).

## 3. Results

### 3.1. Study Selection and Characteristics

**Figure 1:**
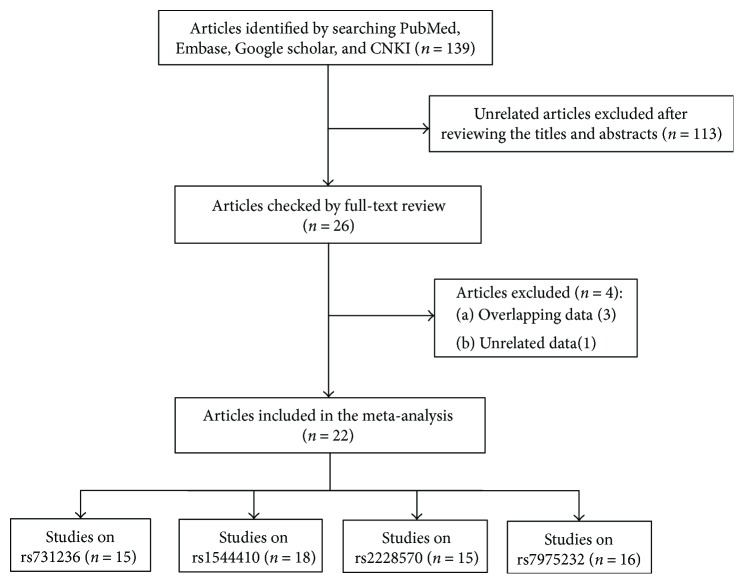
The flowchart for identifying eligible studies.

The flowchart for identifying eligible studies is shown in Figure 1. A total of 139 articles were obtained through an initial search. After reviewing the titles and abstracts of these articles, we excluded 113 unrelated articles. The remaining articles were further checked by a full-text review. Finally, 22 articles met the inclusion criteria and were included in this meta-analysis. The main characteristics of all included articles are shown in Tables 1 and S1. A total of 24 studies from 22 articles assessed the association of *VDR* polymorphisms and AITD risk. Thereinto, there were 15 studies on rs731236, 18 studies on rs1544410, 15 studies on rs2228570, and 16 studies on rs7975232. All studies used polymerase chain reaction-restriction fragment length polymorphism (PCR–RFLP) method for genotyping except for Meng et al.'s study.

### 3.2. Quantitative Synthesis

**Figure 2:**
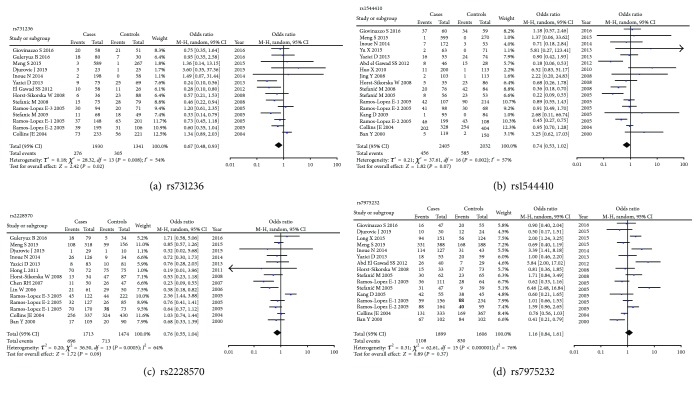
Forest plot of the association of *VDR* polymorphisms with AITD risk in homozygote comparison model.

The association between *VDR* rs731236 polymorphism and AITD risk is shown in Table 2 and Figure 2. In the overall analysis, a significant association was found in homozygote comparison and recessive models (CC versus TT: OR = 0.67, 95% CI: 0.48–0.93, *P*_*Z*_ = 0.02; CC versus CT + TT: OR = 0.80, 95% CI: 0.66–0.95, *P*_*Z*_ = 0.01). In the stratification analysis based on clinical types, a significant association of *VDR* rs731236 polymorphism with HT risk was found in the homozygote comparison model (CC versus TT: OR = 0.58, 95% CI: 0.40–0.85, *P*_*Z*_ = 0.005). In the subgroup analysis by ethnicity, a significant association was found in Asian (CC versus TT: OR = 0.49, 95% CI: 0.28–0.86, *P*_*Z*_ = 0.01) and African populations (CC versus TT: OR = 0.28, 95% CI: 0.10–0.80, *P*_*Z*_ = 0.02; CT versus TT: OR = 0.34, 95% CI: 0.16–0.74, *P*_*Z*_ = 0.007; CC + CT versus TT: OR = 0.33, 95% CI: 0.16–0.86, *P*_*Z*_ = 0.003). The pooled analysis based on *P*_HWE_ > 0.05 showed a significant association in the allele comparison model (C versus T: OR = 0.82, 95% CI: 0.68–0.99, *P*_*Z*_ = 0.04).

The association of *VDR* rs1544410 polymorphism with AITD risk is presented in Table 3 and Figure 2. No significant association was observed in the overall analysis and stratification analysis by clinical types. However, a subgroup analysis by ethnicity showed that *VDR* rs1544410 polymorphism was associated with a reduced risk of AITD in European (AA versus GG: OR = 0.60, 95% CI: 0.46–0.93, *P*_*Z*_ = 0.02; AG versus GG: OR = 0.83, 95% CI: 0.71–0.97, *P*_*Z*_ = 0.02; AA + AG versus GG: OR = 0.79, 95% CI: 0.68–0.91, *P*_*Z*_ = 0.002; A versus G: OR = 0.82, 95% CI: 0.69–0.98, *P*_*Z*_ = 0.02) and African (AA versus GG: OR = 0.18, 95% CI: 0.06–0.53, *P*_*Z*_ = 0.002; AA + AG versus GG: OR = 0.42, 95% CI: 0.20–0.90, *P*_*Z*_ = 0.02; AA versus AG + GG: OR = 0.26, 95% CI: 0.10–0.66, *P*_*Z*_ = 0.005) populations and with an increased risk of AITD in Asian populations (AG versus GG: OR = 1.34, 95% CI: 1.08–1.67, *P*_*Z*_ = 0.008; AA + AG versus GG: OR = 1.41, 95% CI: 1.05–1.90, *P*_*Z*_ = 0.02; A versus G: OR = 1.41, 95% CI: 1.05–1.90, *P*_*Z*_ = 0.02). The pooled analysis based on *P*_HWE_ > 0.05 showed that *VDR* rs1544410 polymorphism was associated with a reduced risk of AITD (AA versus GG: OR = 0.66, 95% CI: 0.45–0.98, *P*_*Z*_ = 0.04).

The association between *VDR* rs2228570 polymorphism and AITD risk is shown in Table 4 and Figure 2. A significant association was observed in the overall analysis (CT versus CC: OR = 0.73, 95% CI: 0.56–0.95, *P*_*Z*_ = 0.02; TT + CT versus CC: OR = 0.71, 95% CI: 0.54–0.93, *P*_*Z*_ < 0.001; T versus C: OR = 0.80, 95% CI: 0.68–0.95, *P*_*Z*_ = 0.01). A further stratification analysis by clinical types showed a significant association in HT (T versus C: OR = 0.69, 95% CI: 0.50–0.97, *P*_*Z*_ = 0.03) but not in GD. A subgroup analysis based on ethnicity showed a significant association in Asian populations (TT versus CC: OR = 0.63, 95% CI: 0.42–0.93, *P*_*Z*_ = 0.02; TT + CT versus CC: OR = 0.65, 95% CI: 0.45–0.95, *P*_*Z*_ = 0.02; TT versus CT + CC: OR = 0.72, 95% CI: 0.58–0.91, *P*_*Z*_ = 0.005; T versus C: OR = 0.72, 95% CI: 0.56–0.92, *P*_*Z*_ = 0.008), but not in European populations. A stratification analysis by *P*_HWE_ value showed a significant association in studies of *P*_H_ ≤ 0.05 (CT versus CC: OR = 0.48, 95% CI: 0.36–0.65, *P*_*Z*_ < 0.001; TT + CT versus CC: OR = 0.51, 95% CI: 0.38–0.68, *P*_*Z*_ < 0.001; T versus C: OR = 0.72, 95% CI: 0.55–0.93, *P*_*Z*_ = 0.01) but not in studies of *P*_H_ > 0.05.

For *VDR* rs7975232 polymorphism, no significant association was observed in the overall analysis and stratification analysis by clinical types and *P*_HWE_ (Table 5 and Figure 2). However, a stratification analysis by ethnicity showed that *VDR* rs7975232 polymorphism was associated with an increased risk of AITD in African populations (CC versus AA: OR = 5.84, 95% CI: 2.00–17.02, *P*_*Z*_ = 0.001; CA versus AA: OR = 3.02, 95% CI: 1.33–6.87, *P*_*Z*_ = 0.008; CC + CA versus AA: OR = 3.62, 95% CI: 1.65–7.93, *P*_*Z*_ = 0.001; CC versus CA + AA: OR = 2.79, 95% CI: 1.12–6.95, *P*_*Z*_ = 0.03; C versus A: OR = 2.29, 95% CI: 1.41–3.73, *P*_*Z*_ < 0.001).

### 3.3. Sensitivity Analysis and Publication Bias

**Figure 3:**
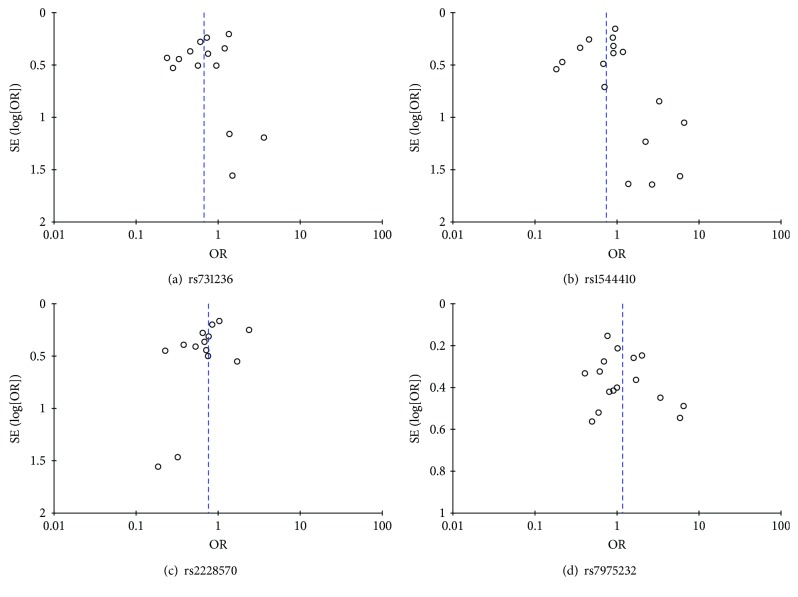
Funnel plot of the association of *VDR* polymorphisms with AITD risk in homozygote comparison model.

A sensitivity analysis showed that the pooled OR values were not substantially changed after one study deletion each time, which suggests that results of this meta-analysis were stable. As shown in Figure 3, the shape of funnel plots was symmetric. In addition, all *P* values of Egger's test were more than 0.05, indicating the lack of publication bias (Table 6).

## 4. Discussion

As an immune modulator, vitamin D is involved in the onset and development of AITD [35, 36]. Low levels of vitamin D have been demonstrated in patients with AITD [36]. Furthermore, vitamin D deficiency was correlated with the duration of HT, which led to an increase in thyroid volume and in antithyroid antibodies levels [36]. Vitamin D exerts its biological effects by binding to VDR and activating VDR-responsive genes [37]. VDR is an intracellular receptor belonging to the steroid/thyroid nuclear receptor family and expressed in human immune cells including macrophages, dendritic cells, and T and B lymphocytes [35]. Therefore, the abnormal function of VDR, which is attributable to *VDR* gene polymorphisms and altered transactivation, might affect the immunoregulatory and anti-inflammatory functions of vitamin D and correlate with the pathogenesis of AITD. Some studies demonstrated that genetic polymorphisms (rs731236, rs1544410, rs2228570, and rs7975232) in the *VDR* gene could affect the expression of VDR [38, 39]. For instance, Ogunkolade et al. found that rs2228570 in the coding region of *VDR* gene was associated with higher VDR mRNA copy numbers [38]. Uitterlinden et al. observed that rs731236, rs1544410, and rs7975232 in the 3′ untranslated region of the *VDR* gene could affect VDR gene expression by modulating mRNA stability [39]. In view of all this, these functional polymorphisms were speculated to be associated with AITD risk. Interestingly, some epidemiological studies confirmed the speculation and found significant association between these polymorphisms and AITD risk. For instance, Long et al. and Meng et al. observed that *VDR* rs7975232 polymorphism was significantly associated with GD risk in Chinese populations [14, 15]. Djurovic et al. found a significant association between *VDR* rs2228570 polymorphism and HT risk in Serbian populations [16]. Stefanić et al. found that *VDR* rs731236, rs7975232, and rs1544410 polymorphisms were associated with GD susceptibility in Eastern Croatian populations [28]. Yazici et al. observed that *VDR* rs731236 and rs2228570 polymorphisms were significantly associated with HT risk in a Turkish population [19]. However, other studies including genome-wide association study showed that these polymorphisms did not influence individual susceptibility to AITD [13, 18, 20, 23, 24, 40, 41]. These inconsistent results may be due to the fact that a single-center pilot study with small sample sizes has low statistical power to detect a true association or that the genetic background of different populations changes the effect of low-penetration polymorphisms on AITD risk. In 2013, Feng et al. tried to clarify the association by meta-analysis and found that *VDR* rs1544410 and rs731236 polymorphisms were significantly associated with a reduced risk of AITD, while *VDR* rs7975232 and rs2228570 polymorphisms were not associated with AITD risk [42]. Due to the limited number of related studies, subgroup analyses by clinical types were not conducted. Furthermore, results of recent studies were still inconsistent with that of previous meta-analysis [13, 15, 16]. Therefore, an updated analysis was performed by combining the recent studies. Results indicated that *VDR* rs731236 and rs2228570 polymorphisms were significantly associated with reduced risk of AITD. A further stratification analysis based on clinical types showed that *VDR* rs731236 and rs2228570 polymorphisms were associated only with reduced risk of HT. In a stratification analysis based on ethnicity, *VDR* rs731236 polymorphism was associated with a reduced risk of AITD in Asian and African populations but not in European populations. *VDR* rs2228570 polymorphism was associated with a reduced risk of AITD only in Asian populations. It was worthy to note that *VDR* rs1544410 polymorphism was associated with a reduced risk of AITD in European and African populations but has an increased risk of AITD in Asian populations. A stratification analysis by *P*_HWE_ values showed that the significant association of *VDR* rs2228570 polymorphism with a reduced risk of AITD was observed only in studies with *P*_HWE_ ≤ 0.05, which indicated that the effect of *VDR* rs2228570 polymorphism on AITD risk needed to be interpreted cautiously.

Heterogeneity was observed in the current meta-analysis. We tried to investigate the sources of heterogeneity by a stratification analysis based on clinical types, ethnicity, and HWE, but the investigation results were not satisfactory and could not provide a reasonable explanation for the sources of heterogeneity. In view of factors affecting vitamin D levels and methodological issues, heterogeneity may result from differences in economic and public health indexes among different countries, variations in environment and climate, age and gender mismatch in published studies, and variations in diagnostic criteria for GD/HT.

Although the current results showed the statistically significant associations of *VDR* polymorphisms with AITD risk, such associations had a small influence on the occurrence of AITD. In addition, several limitations impeding accurate assessment should be noted. Firstly, raw data such as gender, age, living style, and drug consumption could not be obtained from all included studies. Secondly, the number of studies was small in the subgroup analysis, especially in African populations. Last but not least, the present analysis did not consider the gene-gene and gene-environment interactions.

In conclusion, the present study suggested that *VDR* rs731236, rs1544410, rs2228570, and rs7975232 polymorphisms were significantly associated with AITD risk. However, more well-designed studies, especially studies on African populations, should be performed to verify the results.

## Figures and Tables

**Table 1:** The main characteristics of all included articles.

First author	Year of publication	Region	Ethnicity	Cases	Controls	Genotyping method	Polymorphisms
Giovinazzo [13]	2016	Italy	European	100 HT	100	PCR–RFLP	rs731236, rs7975232, rs1544410
Guleryuz [33]	2016	Turkey	Asian	136 HT	50	PCR–RFLP	rs731236, rs2228570
Long [14]	2015	China	Asian	260 GD	221	PCR–RFLP	rs7975232
Meng [15]	2015	China	Asian	417 GD and 250 HT	301	MALDI-TOF-MS	rs731236, rs7975232, rs2228570, rs1544410
Djurovic [16]	2015	Serbia	European	44 HT	32	PCR–RFLP	rs731236, rs7975232, rs2228570
Inoue [17]	2014	Japan	Asian	139 GD and 116 HT	76	PCR–RFLP	rs731236, rs7975232, rs2228570, rs1544410
Yu [18]	2013	China	Asian	75 HT	80	PCR–RFLP	rs1544410
Yazici [19]	2013	Turkey	Asian	111 HT	159	PCR–RFLP	rs731236, rs7975232, rs2228570, rs1544410
El Gawad [34]	2012	Egypt	African	90 GD	55	PCR–RFLP	rs731236, rs7975232, rs1544410
Hong [20]	2011	China	Asian	82 HT	80	PCR–RFLP	rs2228570
Huo [21]	2010	China	Asian	120 GD and 115 HT	120	PCR–RFLP	rs1544410
Horst-Sikorska [22]	2008	Poland	European	75 GD	163	PCR–RFLP	rs731236, rs7975232, rs2228570, rs1544410
Maalej [23]	2008	Tunisia	African	100 AITD	100	PCR–RFLP	rs731236, rs2228570, rs1544410
Jing [24]	2008	China	Asian	115 HT	120	PCR–RFLP	rs1544410
Stefanić [25]	2008	Croatia	European	145 HT	145	PCR–RFLP	rs731236, rs7975232, rs1544410
Chen [31]	2007	Taiwan	Asian	88 GD	90	PCR–RFLP	rs2228570
Lin [26]	2006	Taiwan	Asian	109 HT	90	PCR–RFLP	rs2228570
Ramos-Lopez [27]	2005	Germany, Poland, Serbia	European	789 GD	823	PCR–RFLP	rs731236, rs7975232, rs2228570, rs1544410
Stefanić [28]	2005	Croatia	European	110 GD	99	PCR–RFLP	rs731236, rs7975232, rs1544410
Kang [29]	2005	China	Asian	102 GD	120	PCR–RFLP	rs7975232, rs1544410
Collins [32]	2004	United Kingdom	European	768 GD	864	PCR–RFLP	rs731236, rs7975232, rs2228570, rs1544410
Ban [30]	2000	Japan	Asian	180 GD	195	PCR–RFLP	rs7975232, rs2228570, rs1544410

PCR–RFLP: polymerase chain reaction-restriction fragment length polymorphism; MALDI-TOF-MS: matrix-assisted laser desorption ionization-time of flight mass spectrometry.

**Table 2:** The association between *VDR* rs731236 polymorphism and AITD risk.

Comparison model	Subgroup	The number of studies	Sample size (cases/controls)	*I* ^2^	*P*_H_	Effect model	OR (95% CI)	*P*_*Z*_
Homozygote comparison (CC versus TT)	AITD	14	1930/1341	54%	0.008	Random	**0.67 [0.48, 0.93]**	0.02
	HT	7	625/579	41%	0.12	Fixed	**0.58 [0.40, 0.85]**	0.005
	GD	9	1305/1087	53%	0.03	Random	0.71 [0.49, 1.04]	0.08
	European	9	930/891	53%	0.03	Random	0.75 [0.54, 1.05]	0.09
	Asian	4	942/424	47%	0.13	Fixed	**0.49 [0.28, 0.86]**	0.01
	African	1	58/26	—	—	—	**0.28 [0.10, 0.80]**	0.02
Heterozygote comparison (CT versus TT)	AITD	14	2674/1949	70%	<0.001	Random	0.82 [0.64, 1.05]	0.11
	HT	7	827/778	72%	0.002	Random	0.82 [0.52, 1.29]	0.38
	GD	9	1847/1546	65%	0.004	Random	0.85 [0.65, 1.11]	0.22
	European	9	1471/1354	50%	0.04	Random	0.97 [0.84, 1.13]	0.71
	Asian	4	1123/551	82%	<0.001	Random	0.69 [0.35, 1.35]	0.27
	African	1	80/44	—	—	—	**0.34 [0.16, 0.74]**	0.007
Dominant model (CC + CT versus TT)	AITD	14	2950/2254	75%	<0.001	Random	0.79 [0.61, 1.02]	0.07
	HT	7	895/861	75%	<0.001	Random	0.81 [0.51, 1.29]	0.38
	GD	9	2055/1769	71%	<0.001	Random	0.81 [0.61, 1.07]	0.13
	European	9	1705/1615	62%	0.007	Random	0.90 [0.70, 1.15]	0.39
	Asian	4	1155/584	84%	<0.001	Random	0.69 [0.34, 1.37]	0.29
	African	1	90/55	—	—	—	**0.33 [0.16, 0.68]**	0.003
Recessive model (CC versus CT + TT)	AITD	14	2950/2254	7%	0.37	Fixed	**0.80 [0.66, 0.95]**	0.01
	HT	7	895/861	0%	0.44	Fixed	0.71 [0.50, 1.01]	0.06
	GD	9	2055/1769	6%	0.38	Fixed	0.84 [0.68, 1.03]	0.09
	European	9	1705/1615	26%	0.21	Fixed	0.83 [0.68, 1.01]	0.06
	Asian	4	1155/584	0%	0.61	Fixed	0.67 [0.39, 1.17]	0.16
	African	1	90/55	—	—	—	0.50 [0.20, 1.27]	0.14
Allele comparison (C versus T)	AITD	15	3050/2354	73%	<0.001	Random	0.85 [0.71, 1.02]	0.09
	HT	7	895/861	70%	0.003	Random	0.85 [0.61, 1.19]	0.35
	GD	9	2055/1769	70%	<0.001	Random	0.83 [0.68, 1.02]	0.08
	European	9	1705/1615	66%	0.003	Random	0.89 [0.74, 1.07]	0.21
	Asian	4	1155/584	79%	0.003	Random	0.77 [0.47, 1.26]	0.30
	African	2	190/155	92%	<0.001	Random	0.84 [0.27, 2.54]	0.75
	HWE > 0.05	14	2950/2254	72%	<0.001	Random	**0.82 [0.68, 0.99]**	0.04

*P*_H_: *P* value of heterogeneity test; *P_Z_*: *P* value of *Z* test.

**Table 3:** The association between *VDR* rs1544410 polymorphism and AITD risk.

Comparison model	Subgroup	The number of studies	Sample size (cases/controls)	*I* ^2^	*P*_H_	Effect model	OR (95% CI)	*P*_*Z*_
Homozygote comparison (AA versus GG)	AITD	17	2405/2032	57%	0.002	Random	0.74 [0.53, 1.02]	0.07
	HT	8	763/837	34%	0.15	Fixed	0.80 [0.55, 1.16]	0.24
	GD	12	1642/1631	68%	<0.001	Random	0.71 [0.46, 1.09]	0.11
	European	8	959/1076	65%	0.005	Random	**0.60 [0.46, 0.93]**	0.02
	Asian	8	1400/928	0%	0.47	Fixed	1.51 [0.90, 2.55]	0.12
	African	1	46/28	—	—	—	**0.18 [0.06, 0.53]**	0.002
	*P*_HWE_ > 0.05	13	1823/1539	62%	0.001	Random	**0.66 [0.45, 0.98]**	0.04
	*P*_HWE_ ≤ 0.05	4	582/493	28%	0.24	Fixed	1.06 [0.70, 1.61]	0.79
Heterozygote comparison (AG versus GG)	AITD	17	3180/2788	38%	0.05	Random	0.99 [0.84, 1.18]	0.93
	HT	8	925/984	0%	0.63	Fixed	1.07 [0.84, 1.36]	0.61
	GD	12	2255/2285	60%	0.004	Random	0.99 [0.78, 1.25]	0.94
	European	8	1432/1638	0%	0.85	Fixed	**0.83 [0.71, 0.97]**	0.02
	Asian	8	1666/1110	22%	0.25	Fixed	**1.34 [1.08, 1.67]**	0.008
	African	1	82/40	—	—	—	0.56 [0.25, 1.23]	0.15
	*P*_HWE_ > 0.05	13	2475/2103	22%	0.22	Fixed	0.95 [0.82, 1.08]	0.42
	*P*_HWE_ ≤ 0.05	4	705/685	71%	0.01	Random	1.32 [0.69, 2.54]	0.41
Dominant model (AA + AG versus GG)	AITD	17	3636/3373	61%	<0.001	Random	0.98 [0.80, 1.20]	0.82
	HT	8	1009/1089	25%	0.23	Fixed	1.03 [0.82, 1.29]	0.82
	GD	12	2627/2769	73%	<0.001	Random	0.97 [0.74, 1.27]	0.81
	European	8	1835/2177	3%	0.41	Fixed	**0.79 [0.68, 0.91]**	0.002
	Asian	8	1711/1141	44%	0.09	Random	**1.41 [1.05, 1.90]**	0.02
	African	1	90/55	—	—	—	**0.42 [0.20, 0.90]**	0.02
	*P*_HWE_ > 0.05	13	2869/2593	56%	0.008	Random	0.91 [0.74, 1.12]	0.38
	*P*_HWE_ ≤ 0.05	4	767/780	77%	0.004	Random	1.39 [0.70, 2.76]	0.34
Recessive model (AA versus AG + GG)	AITD	17	3636/3373	58%	0.002	Random	0.79 [0.59, 1.06]	0.11
	HT	8	1009/1089	35%	0.15	Fixed	0.82 [0.59, 1.13]	0.22
	GD	12	2627/2769	67%	<0.001	Random	0.77 [0.53, 1.12]	0.17
	European	8	1835/2177	71%	0.001	Random	0.74 [0.53, 1.02]	0.06
	Asian	8	1711/1141	0%	0.57	Fixed	1.42 [0.87, 2.33]	0.16
	African	1	90/55	—	—	—	**0.26 [0.10, 0.66]**	0.005
	*P*_HWE_ > 0.05	13	2869/2593	63%	0.001	Random	0.72 [0.51, 1.01]	0.05
	*P*_HWE_ ≤ 0.05	4	767/780	8%	0.35	Fixed	1.18 [0.82, 1.72]	0.37
Allele comparison (A versus G)	AITD	18	3736/3473	72%	<0.001	Random	0.96 [0.81, 1.13]	0.63
	HT	8	1009/1089	58%	0.02	Random	1.08 [0.81, 1.44]	0.60
	GD	12	2627/2769	80%	<0.001	Random	0.97 [0.77, 1.21]	0.76
	European	8	1835/2177	66%	0.005	Random	**0.82 [0.69, 0.98]**	0.02
	Asian	8	1711/1141	58%	0.02	Random	**1.41 [1.05, 1.90]**	0.02
	African	2	190/155	72%	0.06	Random	0.64 [0.35, 1.15]	0.14
	*P*_HWE_ > 0.05	13	2969/2693	70%	<0.001	Random	0.89 [0.75, 1.06]	0.21
	*P*_HWE_ ≤ 0.05	4	767/780	79%	0.002	Random	1.44 [0.78, 2.65]	0.24

*P*_H_: *P* value of heterogeneity test; *P_Z_*: *P* value of *Z* test.

**Table 4:** The association between *VDR* rs2228570 polymorphism and AITD risk.

Comparison model	Subgroup	The number of studies	Sample size (cases/controls)	*I* ^2^	*P*_H_	Effect model	OR (95% CI)	*P*_*Z*_
Homozygote comparison (TT versus CC)	AITD	14	1713/1474	64%	<0.001	Random	0.76 [0.55, 1.04]	0.09
	HT	7	509/440	27%	0.22	Fixed	0.77 [0.55, 1.07]	0.11
	GD	9	1204/1224	73%	<0.001	Random	0.79 [0.54, 1.15]	0.22
	European	6	819/907	72%	0.003	Random	0.93 [0.58, 1.49]	0.76
	Asian	8	894/567	45%	0.08	Random	**0.63 [0.42, 0.93]**	0.02
	*P*_HWE_ > 0.05	11	1409/1301	71%	<0.001	Random	0.78 [0.53, 1.14]	0.20
	*P*_HWE_ ≤ 0.05	3	304/173	0%	0.89	Fixed	0.65 [0.42, 1.00]	0.05
Heterozygote comparison (CT versus CC)	AITD	14	2478/2123	72%	<0.001	Random	**0.73 [0.56, 0.95]**	0.02
	HT	7	665/600	77%	0.001	Random	0.61 [0.35, 1.06]	0.08
	GD	9	1813/1832	65%	0.004	Random	0.83 [0.64, 1.07]	0.14
	European	6	1145/1315	77%	<0.001	Random	0.78 [0.52, 1.16]	0.22
	Asian	8	1333/808	70%	0.001	Random	0.68 [0.47, 1.00]	0.05
	*P*_HWE_ > 0.05	11	2008/1762	66%	0.001	Random	0.84 [0.64, 1.10]	0.21
	*P*_HWE_ ≤ 0.05	3	470/361	32%	0.23	Fixed	**0.48 [0.36, 0.65]**	<0.001
Dominant model (TT + CT versus CC)	AITD	14	3174/2836	76%	<0.001	Random	**0.71 [0.54, 0.93]**	<0.001
	HT	7	839/788	77%	<0.001	Random	0.60 [0.35, 1.02]	0.06
	GD	9	2335/2425	72%	<0.001	Random	0.81 [0.62, 1.06]	0.12
	European	6	1562/1795	80%	<0.001	Random	0.78 [0.52, 1.18]	0.24
	Asian	8	1612/1041	72%	<0.001	Random	**0.65 [0.45, 0.95]**	0.02
	*P*_HWE_ > 0.05	11	2616/2416	74%	<0.001	Random	0.81 [0.60, 1.08]	0.14
	*P*_HWE_ ≤ 0.05	3	558/420	36%	0.21	Fixed	**0.51 [0.38, 0.68]**	<0.001
Recessive model (TT versus CT + CC)	AITD	14	3174/2836	54%	0.008	Random	0.87 [0.68, 1.10]	0.23
	HT	7	839/788	0%	0.46	Fixed	0.79 [0.59, 1.06]	0.11
	GD	9	2335/2425	64%	0.004	Random	0.90 [0.68, 1.19]	0.46
	European	6	1562/1795	60%	0.03	Random	1.05 [0.76, 1.45]	0.79
	Asian	8	1612/1041	23%	0.24	Fixed	**0.72 [0.58, 0.91]**	0.005
	*P*_HWE_ > 0.05	11	2616/2416	64%	0.002	Random	0.83 [0.62, 1.11]	0.20
	*P*_HWE_ ≤ 0.05	3	558/420	0%	0.89	Fixed	1.02 [0.71, 1.48]	0.91
Allele comparison (T versus C)	AITD	15	3274/2936	75%	<0.001	Random	**0.80 [0.68, 0.95]**	0.01
	HT	7	839/788	72%	0.001	Random	**0.69 [0.50, 0.97]**	0.03
	GD	9	2335/2425	76%	<0.001	Random	0.87 [0.73, 1.05]	0.15
	European	6	1562/1795	79%	<0.001	Random	0.90 [0.70, 1.16]	0.41
	Asian	8	1612/1041	69%	0.002	Random	**0.72 [0.56, 0.92]**	0.008
	African	1	100/100	—	—	—	0.86 [0.53, 1.39]	0.54
	*P*_HWE_ > 0.05	11	2716/2516	77%	<0.001	Random	0.83 [0.68, 1.01]	0.06
	*P*_HWE_ ≤ 0.05	3	558/420	38%	0.20	Fixed	**0.72 [0.55, 0.93]**	0.01

*P*_H_: *P* value of heterogeneity test; *P_Z_*: *P* value of *Z* test.

**Table 5:** The association between *VDR* rs7975232 polymorphism and AITD risk.

Comparison model	Subgroup	The number of studies	Sample size (cases/controls)	*I* ^2^	*P*_H_	Effect model	OR (95% CI)	*P*_*Z*_
Homozygote comparison (CC versus AA)	AITD	16	1899/1606	76%	<0.001	Random	1.16 [0.84, 1.61]	0.37
	HT	6	396/434	33%	0.19	Fixed	1.11 [0.80, 1.55]	0.52
	GD	12	1503/1403	81%	<0.001	Random	1.22 [0.81, 1.83]	0.34
	European	9	983/1016	71%	<0.001	Random	1.10 [0.76, 1.60]	0.62
	Asian	6	876/561	80%	<0.001	Random	1.02 [0.55, 1.92]	0.94
	African	1	40/29	—	—	—	**5.84 [2.00, 17.02]**	0.001
	*P*_HWE_ > 0.05	12	1714/1439	76%	<0.001	Random	1.14 [0.81, 1.61]	0.45
	*P*_HWE_ ≤ 0.05	4	185/167	82%	0.001	Random	1.19 [0.39, 3.64]	0.75
Heterozygote comparison (CA versus AA)	AITD	16	2436/2287	61%	<0.001	Random	1.04 [0.83, 1.31]	0.71
	HT	6	504/538	30%	0.21	Fixed	1.11 [0.84, 1.47]	0.45
	GD	12	1932/1926	67%	<0.001	Random	1.03 [0.79, 1.36]	0.81
	European	9	1468/1585	43%	0.08	Random	1.02 [0.82, 1.27]	0.86
	Asian	6	904/654	71%	0.004	Random	0.91 [0.56, 1.47]	0.70
	African	1	64/48	—	—	—	**3.02 [1.33, 6.87]**	0.008
	*P*_HWE_ > 0.05	12	2152/1976	66%	<0.001	Random	1.06 [0.83, 1.37]	0.63
	*P*_HWE_ ≤ 0.05	4	284/311	55%	0.09	Random	0.96 [0.53, 1.75]	0.90
Dominant model (CC + CA versus AA)	AITD	16	3544/3117	73%	<0.001	Random	1.08 [0.84, 1.38]	0.56
	HT	6	757/812	37%	0.16	Fixed	1.08 [0.83, 1.40]	0.56
	GD	12	2787/2681	78%	<0.001	Random	1.09 [0.80, 1.49]	0.57
	European	9	1884/2011	61%	0.009	Random	1.04 [0.81, 1.34]	0.73
	Asian	6	1570/1051	79%	<0.001	Random	0.94 [0.55, 1.60]	0.81
	African	1	90/55	—	—	—	**3.62 [1.65, 7.93]**	0.001
	*P*_HWE_ > 0.05	12	3159/2727	75%	<0.001	Random	1.10 [0.84, 1.45]	0.50
	*P*_HWE_ ≤ 0.05	4	385/390	71%	0.02	Random	0.97 [0.48, 1.96]	0.94
Recessive model (CC versus CA + AA)	AITD	16	3544/3117	59%	0.001	Random	1.10 [0.90, 1.33]	0.36
	HT	6	757/812	18%	0.29	Fixed	0.97 [0.77, 1.21]	0.78
	GD	12	2787/2681	67%	<0.001	Random	1.13 [0.89, 1.42]	0.31
	European	9	1884/2011	58%	0.02	Random	1.06 [0.81, 1.40]	0.65
	Asian	6	1570/1051	62%	0.02	Random	1.06 [0.79, 1.42]	0.71
	African	1	90/55	—	—	—	**2.79 [1.12, 6.95]**	0.03
	*P*_HWE_ > 0.05	12	3159/2727	52%	0.02	Random	1.06 [0.87, 1.28]	0.57
	*P*_HWE_ ≤ 0.05	4	385/390	77%	0.005	Random	1.25 [0.58, 2.68]	0.57
Allele comparison (C versus A)	AITD	16	3544/3117	75%	<0.001	Random	1.06 [0.91, 1.24]	0.44
	HT	6	757/812	33%	0.19	Fixed	1.01 [0.87, 1.17]	0.88
	GD	12	2787/2681	81%	<0.001	Random	1.09 [0.90, 1.32]	0.37
	European	9	1884/2011	70%	<0.001	Random	1.04 [0.86, 1.25]	0.69
	Asian	6	1570/1051	78%	<0.001	Random	1.00 [0.77, 1.31]	0.98
	African	1	90/55	—	—	—	**2.29 [1.41, 3.73]**	<0.001
	*P*_HWE_ > 0.05	12	3159/2727	76%	<0.001	Random	1.06 [0.90, 1.25]	0.47
	*P*_HWE_ ≤ 0.05	4	385/390	79%	0.002	Random	1.04 [0.65, 1.66]	0.88

*P*_H_: *P* value of heterogeneity test; *P_Z_*: *P* value of *Z* test.

**Table 6:** Egger's test results for the publication bias of *VDR* polymorphisms and AITD risk.

Comparison model	*P* value of Egger's test			
	rs731236	rs1544410	rs2228570	rs7975232
Homozygote comparison	0.516	0.626	0.125	0.258
Heterozygote comparison	0.271	0.521	0.07	0.274
Dominant model	0.287	0.444	0.07	0.283
Recessive model	0.381	0.933	0.092	0.130
Allele comparison	0.307	0.422	0.062	0.186

## Supplementary Materials

Supplementary Materials Table S1: genotype and allele frequency distributions of *VDR* polymorphisms in all included studies.
