# Supplementary material for: Meta-Analysis of the Association between Vitamin D Receptor Polymorphisms and the Risk of Autoimmune Thyroid Disease
Source: Int J Endocrinol. 2018 Mar 22;2018:2846943. doi: 10.1155/2018/2846943 (PMC5885334; doi:10.1155/2018/2846943)
Supplement: Supplementary Materials — Table S1: genotype and allele frequency distributions of VDR polymorphisms in all included studies. [file 2846943.f1.docx]

Table S1. Genotype and allele frequency distributions of *VDR* polymorphisms in all included studies

| **Polymorphisms** |  |  | **Cases** | | | | | |  | **Controls** | | | | | |  |
| --- | --- | --- | --- | --- | --- | --- | --- | --- | --- | --- | --- | --- | --- | --- | --- | --- |
| rs731236 | First author | Year of publication | TT | TC | CC | T | C | Total |  | TT | TC | CC | T | C | Total | *P*_HWE_ |
|  | Giovinazzo S | 2016 | 38 | 42 | 20 | 118 | 82 | 100 |  | 30 | 49 | 21 | 109 | 91 | 100 | 0.905 |
|  | Guleryuz B | 2016 | 62 | 56 | 18 | 180 | 92 | 136 |  | 23 | 19 | 7 | 65 | 33 | 49 | 0.356 |
|  | Meng S | 2015 | 586 | 78 | 3 | 1250 | 84 | 667 |  | 266 | 34 | 1 | 566 | 36 | 301 | 0.938 |
|  | Djurovic J | 2015 | 20 | 14 | 3 | 54 | 20 | 37 |  | 24 | 7 | 1 | 55 | 9 | 32 | 0.591 |
|  | Inoue N | 2014 | 196 | 43 | 2 | 435 | 47 | 241 |  | 58 | 17 | 0 | 133 | 17 | 75 | 0.268 |
|  | Yazici D | 2013 | 66 | 36 | 9 | 168 | 54 | 111 |  | 44 | 90 | 25 | 178 | 140 | 159 | 0.061 |
|  | Abd El Gawad SS | 2012 | 48 | 32 | 10 | 128 | 52 | 90 |  | 15 | 29 | 11 | 59 | 51 | 55 | 0.656 |
|  | Horst-Sikorska W | 2008 | 30 | 39 | 6 | 99 | 51 | 75 |  | 65 | 75 | 23 | 205 | 121 | 163 | 0.855 |
|  | Stefanić M | 2008 | 60 | 70 | 15 | 190 | 100 | 145 |  | 51 | 66 | 28 | 168 | 122 | 145 | 0.426 |
|  | Ramos-Lopez E-1 | 2005 | 111 | 100 | 37 | 322 | 174 | 248 |  | 138 | 152 | 63 | 428 | 278 | 353 | 0.065 |
|  | Ramos-Lopez E-2 | 2005 | 156 | 144 | 39 | 456 | 222 | 339 |  | 75 | 79 | 31 | 229 | 141 | 185 | 0.198 |
|  | Ramos-Lopez E-3 | 2005 | 64 | 100 | 30 | 228 | 160 | 194 |  | 51 | 68 | 20 | 170 | 108 | 139 | 0.727 |
|  | Stefanić M | 2005 | 57 | 42 | 11 | 156 | 64 | 110 |  | 31 | 50 | 18 | 112 | 86 | 99 | 0.782 |
|  | Collins JE | 2004 | 160 | 224 | 73 | 544 | 370 | 457 |  | 165 | 178 | 56 | 508 | 290 | 399 | 0.474 |
|  | Maalej A | 2008 | NA | NA | NA | 123 | 77 | 100 |  | NA | NA | NA | 140 | 60 | 100 | NA |
| rs1544410 | First author | Year of publication | AA | AG | GG | A | G | Total |  | AA | AG | GG | A | G | Total | *P*_HWE_ |
|  | Giovinazzo S | 2016 | 37 | 40 | 23 | 114 | 86 | 100 |  | 34 | 41 | 25 | 109 | 91 | 100 | 0.083 |
|  | Meng S | 2015 | 1 | 72 | 594 | 74 | 1260 | 667 |  | 0 | 31 | 270 | 31 | 571 | 301 | 0.346 |
|  | Inoue N | 2014 | 7 | 36 | 165 | 50 | 366 | 208 |  | 3 | 11 | 50 | 17 | 111 | 64 | 0.042 |
|  | Yu X | 2013 | 2 | 12 | 61 | 16 | 134 | 75 |  | 0 | 9 | 71 | 9 | 151 | 80 | 0.594 |
|  | Yazici D | 2013 | 16 | 58 | 37 | 90 | 132 | 111 |  | 24 | 85 | 50 | 133 | 185 | 159 | 0.214 |
|  | Abd El Gawad SS | 2012 | 8 | 44 | 38 | 60 | 120 | 90 |  | 15 | 27 | 13 | 57 | 53 | 55 | 0.9 |
|  | Huo X | 2010 | 11 | 35 | 189 | 57 | 413 | 235 |  | 1 | 7 | 112 | 9 | 231 | 120 | 0.036 |
|  | Horst-Sikorska W | 2008 | 7 | 40 | 28 | 54 | 96 | 75 |  | 23 | 77 | 63 | 123 | 203 | 163 | 0.946 |
|  | Jing Y | 2008 | 2 | 12 | 101 | 16 | 214 | 115 |  | 1 | 7 | 112 | 9 | 231 | 120 | 0.036 |
|  | Stefanić M | 2008 | 20 | 69 | 56 | 109 | 181 | 145 |  | 42 | 61 | 42 | 145 | 145 | 145 | 0.056 |
|  | Ramos-Lopez E-1 | 2005 | 42 | 102 | 65 | 186 | 232 | 209 |  | 90 | 262 | 124 | 442 | 510 | 476 | 0.02 |
|  | Ramos-Lopez E-2 | 2005 | 46 | 138 | 153 | 230 | 444 | 337 |  | 43 | 86 | 65 | 172 | 216 | 194 | 0.156 |
|  | Ramos-Lopez E-3 | 2005 | 41 | 113 | 57 | 195 | 227 | 211 |  | 30 | 85 | 38 | 145 | 161 | 153 | 0.158 |
|  | Stefanić M | 2005 | 8 | 54 | 48 | 70 | 150 | 110 |  | 23 | 46 | 30 | 92 | 106 | 99 | 0.511 |
|  | Kang D | 2005 | 1 | 25 | 94 | 27 | 213 | 120 |  | 0 | 18 | 84 | 18 | 186 | 102 | 0.328 |
|  | Collins JE | 2004 | 202 | 320 | 126 | 724 | 572 | 648 |  | 254 | 443 | 150 | 697 | 743 | 847 | 0.071 |
|  | Ban Y | 2000 | 5 | 61 | 114 | 71 | 289 | 180 |  | 2 | 45 | 148 | 49 | 341 | 195 | 0.482 |
|  | Maalej A | 2008 | NA | NA | NA | 109 | 91 | 100 |  | NA | NA | NA | 117 | 83 | 100 | NA |
| rs2228570 | First author | Year of publication | CC | CT | TT | C | T | Total |  | CC | CT | TT | C | T | Total | *P*_HWE_ |
|  | Guleryuz B | 2016 | 61 | 57 | 18 | 179 | 93 | 136 |  | 29 | 16 | 5 | 74 | 26 | 50 | 0.234 |
|  | Meng S | 2015 | 210 | 349 | 108 | 769 | 565 | 667 |  | 97 | 145 | 59 | 339 | 263 | 301 | 0.716 |
|  | Djurovic J | 2015 | 28 | 15 | 1 | 71 | 17 | 44 |  | 9 | 22 | 1 | 40 | 24 | 32 | 0.008 |
|  | Inoue N | 2014 | 100 | 113 | 26 | 313 | 165 | 239 |  | 25 | 42 | 9 | 92 | 60 | 76 | 0.172 |
|  | Yazici D | 2013 | 75 | 28 | 8 | 178 | 44 | 111 |  | 71 | 78 | 10 | 220 | 98 | 159 | 0.058 |
|  | Hong L | 2011 | 2 | 10 | 70 | 14 | 150 | 82 |  | 0 | 5 | 75 | 5 | 155 | 80 | 0.773 |
|  | Horst-Sikorska W | 2008 | 21 | 41 | 13 | 83 | 67 | 75 |  | 40 | 76 | 47 | 156 | 170 | 163 | 0.401 |
|  | Chen RH | 2007 | 39 | 38 | 11 | 116 | 60 | 88 |  | 21 | 43 | 26 | 85 | 95 | 90 | 0.694 |
|  | Lin W | 2006 | 40 | 48 | 21 | 128 | 90 | 109 |  | 21 | 40 | 29 | 82 | 98 | 90 | 0.324 |
|  | Ramos-Lopez E-1 | 2005 | 77 | 111 | 45 | 265 | 201 | 233 |  | 178 | 188 | 44 | 544 | 276 | 410 | 0.588 |
|  | Ramos-Lopez E-2 | 2005 | 100 | 164 | 70 | 364 | 304 | 334 |  | 35 | 120 | 38 | 190 | 196 | 193 | 0.001 |
|  | Ramos-Lopez E-3 | 2005 | 95 | 95 | 32 | 285 | 159 | 222 |  | 59 | 76 | 26 | 194 | 128 | 161 | 0.854 |
|  | Collins JE | 2004 | 81 | 317 | 256 | 479 | 829 | 654 |  | 106 | 406 | 324 | 618 | 1054 | 836 | 0.223 |
|  | Ban Y | 2000 | 88 | 75 | 17 | 251 | 109 | 180 |  | 70 | 105 | 20 | 245 | 145 | 195 | 0.033 |
|  | Maalej A | 2008 | NA | NA | NA | 161 | 39 | 100 |  | NA | NA | NA | 156 | 44 | 100 | NA |
| rs7975232 | First author | Year of publication | AA | CA | CC | A | C | Total |  | AA | CA | CC | A | C | Total | *P*_HWE_ |
|  | Giovinazzo S | 2016 | 31 | 53 | 16 | 115 | 85 | 100 |  | 35 | 45 | 20 | 115 | 85 | 100 | 0.428 |
|  | Long X | 2015 | 57 | 109 | 94 | 223 | 297 | 260 |  | 68 | 97 | 56 | 233 | 209 | 221 | 0.075 |
|  | Meng S | 2015 | 57 | 279 | 331 | 393 | 941 | 667 |  | 20 | 113 | 168 | 153 | 449 | 301 | 0.865 |
|  | Djurovic J | 2015 | 20 | 14 | 10 | 54 | 34 | 44 |  | 12 | 8 | 12 | 32 | 32 | 32 | 0.005 |
|  | Inoue N | 2014 | 13 | 105 | 114 | 131 | 333 | 232 |  | 12 | 32 | 31 | 56 | 94 | 75 | 0.445 |
|  | Yazici D | 2013 | 35 | 58 | 18 | 128 | 94 | 111 |  | 39 | 100 | 20 | 178 | 140 | 159 | <0.001 |
|  | Abd El Gawad SS | 2012 | 14 | 50 | 26 | 78 | 102 | 90 |  | 22 | 26 | 7 | 70 | 40 | 55 | 0.874 |
|  | Horst-Sikorska W | 2008 | 18 | 42 | 15 | 78 | 72 | 75 |  | 36 | 90 | 37 | 162 | 164 | 163 | 0.183 |
|  | Stefanić M | 2008 | 32 | 83 | 30 | 147 | 143 | 145 |  | 42 | 80 | 23 | 164 | 126 | 145 | 0.139 |
|  | Ramos-Lopez E-1 | 2005 | 97 | 104 | 59 | 298 | 222 | 260 |  | 146 | 196 | 88 | 488 | 372 | 430 | 0.138 |
|  | Ramos-Lopez E-2 | 2005 | 76 | 135 | 88 | 287 | 311 | 299 |  | 55 | 70 | 40 | 180 | 150 | 165 | 0.064 |
|  | Ramos-Lopez E-3 | 2005 | 75 | 84 | 36 | 234 | 156 | 195 |  | 36 | 62 | 28 | 134 | 118 | 126 | 0.894 |
|  | Stefanić M | 2005 | 16 | 63 | 31 | 95 | 125 | 110 |  | 30 | 60 | 9 | 120 | 78 | 99 | 0.007 |
|  | Kang D | 2005 | 13 | 65 | 42 | 91 | 149 | 120 |  | 7 | 55 | 38 | 69 | 131 | 100 | 0.03 |
|  | Collins JE | 2004 | 202 | 323 | 131 | 727 | 585 | 656 |  | 198 | 384 | 169 | 780 | 722 | 751 | 0.508 |
|  | Ban Y | 2000 | 35 | 78 | 67 | 148 | 212 | 180 |  | 18 | 93 | 84 | 129 | 261 | 195 | 0.281 |
